# Characterization of Successful Patterns of Play in Field Hockey from Wearable Sensor Data

**DOI:** 10.3390/s26216965

**Published:** 2026-11-04

**Authors:** Trent Piccicacco, Brendyn Appleby, Dale W. Chapman, Simon Rosalie, Kevin J. Netto, Ritu Gupta

**Affiliations:** 1 School of Electrical Engineering, Computing and Mathematical Sciences, Curtin University, Perth WA 6102, Australia; trent_piccicacco@hotmail.com (T.P.); r.gupta@curtin.edu.au (R.G.); 2 Hockey Australia, Parkville VIC 3052, Australia; brendyn.appleby@murdoch.edu.au; 3 School of Allied Health, Murdoch University, Perth WA 6150, Australia; 4 Curtin School of Allied Health, Curtin University, Perth WA 6102, Australia; dale.chapman@curtin.edu.au; 5 SR Performance, Melbourne VIC 3199, Australia; simon@srperformance.au

**Keywords:** team sport, performance analysis, GPS tracking, random forest, spatiotemporal features, machine learning

## Abstract

**Highlights:**

**What is the main finding?**

• Successful attacking plays were characterized by specific, quantifiable movement patterns which are identifiably different from unsuccessful plays: player closeness (i.e., maintaining ~8–15 m of spacing), player movement (acceleration above 2.5 m·s^−2^), and nine or more players actively involved within the 23 m area.

**What are the implications of the main findings?**

• Coaches and performance analysts can move away from purely subjective tactical assessments and instead use objective, data-driven metrics to evaluate and design attacking plays, providing a measurable basis for training decisions and in-game strategy.
• The computational framework developed in this study is not limited to field hockey, as any team sport where GPS tracking captures player movement can potentially adopt this methodology, opening broader applications across competitive sport for spatial–temporal pattern recognition and performance optimization.

**Abstract:**

Analyzing patterns of play in team sports remains challenging despite advances in GPS tracking technology. While individual player statistics are well-documented, understanding what makes specific team plays successful requires extracting meaningful features from spatial–temporal data. This study addresses the question: can machine learning algorithms effectively characterize successful versus unsuccessful attacking plays in field hockey based on GPS-derived team movement patterns? We analyzed GPS data from a national men’s field hockey team recorded during the 2018 Men’s Hockey World Cup organized by the International Hockey Federation (FIH). We developed novel team-based features from longitude and latitude coordinates, including player closeness (~8–15 m Inter-Player spacing), corner positioning, movement energy via discrete wavelet transforms, and velocity–acceleration metrics. Thirteen features describing 63 attacking plays (42 successful, 21 unsuccessful) were classified using Random Forest (RF). Success was defined as achieving a goal, shot on goal, or attacking circle entry. Using 5-fold cross-validation repeated 10 times, the Random Forest model achieved a mean classification accuracy of 78% (95% CI: 75–81%) and an AUC of 0.88 (95%CI: 0.85–0.91). Sensitivity analyses showed that removing Opposition had minimal impact on predictive performance (accuracy 77.1%, AUC 0.862), while leave-one-match-out cross-validation yielded variable performance across held-out matches (accuracy 60–82%, AUC 0.69–0.96). The most discriminative features were player closeness during plays, high maximum acceleration (>2.5 m·s^−2^), number of athletes involved (9+ players), and mean distance traveled (~10 m). Play duration was also critical, with successful plays lasting 3–6 s. Machine learning can effectively characterize successful field hockey plays from GPS data when appropriate team-oriented features are extracted. Although the novel wavelet-based player energy metric showed a negative predictive contribution in its current form, it may still show promise for future load management applications. This computational methodological framework is potentially generalizable to other team sports requiring spatial–temporal pattern recognition.

## 1. Introduction

Predictive analytics in sport varies widely between contexts, with major advances in analyzing in-play performance indicators for coaching and enhanced decision making. Yet, despite extensive research on individual metrics, few studies have examined successful team plays using explicitly team-based indicators [1]. This is a key gap, as team sport success arises mainly from coordinated collective behavior rather than isolated actions. In field hockey, where 11 players interact across a 91.4 m × 55 m pitch, identifying recurrent successful patterns of coordinated play could yield substantial tactical benefits. Computational methods that quantify and classify such team-level behaviors can therefore move performance analysis beyond traditional individual-focused metrics [2,3]. Field hockey set pieces, particularly short and long corners, are especially suited to this work: they are discrete, have clear start and end points, produce unambiguous outcomes, and rely on tightly coordinated team movements. They also generate rich spatiotemporal data via GPS tracking. However, converting raw longitude–latitude data into tactical insight requires sophisticated, sport-specific feature engineering and domain-informed computational methods.

Previous research has established frameworks for analyzing sports through various computational lenses [4]. Video-based analysis systems and notational coding have proven effective for identifying patterns of play and extracting individual player statistics [5,6,7]. Spatial–temporal analyses in soccer have examined how space around players influences movement decisions [5,6,7], while heat maps have visualized individual player positioning, though with acknowledged bias toward individual rather than team-scale insights [5,6,7]. Temporal pattern (T-pattern) analysis has identified complex structural regularities in soccer coaching tactics [8], though without explicit classification of play success. In basketball, self-organizing maps have been applied to spatial–temporal data for play-type identification [9], demonstrating potential for machine learning in team sports analytics.

Despite these advances, a critical gap remains, with very few studies having attempted to classify specific sporting plays as successful or unsuccessful using team-level features derived from GPS tracking data. Machine learning classification algorithms—Support Vector Machines (SVM), Linear Discriminant Analysis (LDA), and Random Forests—offer powerful tools for such binary classification problems [10]. SVM focuses on difficult-to-classify observations by constructing hyperplanes that maximize class separation [11]. LDA transforms multidimensional data to lower dimensions that maximize between-class variance, assuming normality and equal covariances [12]. Random Forests utilize ensembles of decision trees with bagging to reduce overfitting and handle nonlinear relationships effectively, which is particularly valuable for small datasets [13,14,15].

The main challenge is not the classification algorithms but feature engineering, that is, deriving variables that capture team dynamics from raw GPS coordinates. Distances between players, movement patterns, acceleration profiles, and positional relationships must become quantitative features that retain tactical meaning. Without well-designed features that explain variation in play outcomes, even advanced algorithms perform poorly. This study therefore sought to (1) develop novel features from GPS tracking data reflecting field hockey team behaviors, (2) identify behavioral features that best predict play success, and (3) convert computational results into practical coaching recommendations. We hypothesized that team-level spatial features, such as player clustering and coordinated movement, would provide greater explanatory value for successful plays than individual-level metrics, reflecting the collective and dynamic nature of team performance.

## 2. Materials and Methods

### 2.1. Data Collection and Study Design

This retrospective analysis used de-identified performance data collected during an international competition as part of routine athlete monitoring conducted by the national sporting organization. Data were provided through an institutional collaboration between the sporting organization and the University. When ethical clearance for the study was submitted, the Institutional Human Research Ethics Committee ruled that no ethical clearance was required, as the data were de-identified and footage used for coding was open source and freely accessible. GPS tracking data were provided by the national sporting organization of the national men’s field hockey team during the 2018 Men’s Hockey World Cup organized by the International Hockey Federation (FIH). Data were collected at 10 Hz using OptimEye S5 (Catapult, Melbourne, Australia) GPS units worn by all field players during matches against five opponent national men’s teams (Ireland, France, England, China, and the Netherlands). These devices have previously been reported to have suitable reliability and validity [16,17]. The commercial provider’s proprietary filtering was automatically applied, and each unit recorded timestamp, velocity (m·s^−1^) (differentiated position), acceleration (m·s^−2^) (double differentiation), cumulative distance (odometer), latitude, and longitude coordinates. No internal physiological measures were recorded during these matches.

A research assistant manually annotated play timestamps by reviewing game footage, identifying 65 attacking corner plays (short corners and long corners). Play duration was defined from the start of the attacking corner sequence until the attacking chain of play concluded or play was stopped by the umpire’s whistle. Thus, play duration was not a prospective predictor, as the rules of the game do not impose a shot or attacking time constraint. Each play was classified by outcome: success (3 = goal scored, 2 = shot on goal, 1 = circle entry) or failure (0 = no scoring opportunity created). Because short corners begin with the ball injected into the circle by design, circle entry alone was not treated as a successful outcome for these plays; instead, the lowest success level (1) required the attacking team to establish a controlled attacking set play within the circle beyond the initial injection. For long corners, taken from outside the circle, all three success levels reflected genuine attacking progression. Two plays (one successful and one unsuccessful) were excluded from the analysis due to timestamp data quality issues that resulted in implausible duration measurements. This resulted in a final sample of 63 plays, comprising 42 successful and 21 unsuccessful plays.

Across the 42 successful plays, outcomes were distributed as goal scored (n = 16), shot on goal (n = 16), and circle entry (n = 10); the remaining 22 plays were coded as failures. Additional contextual variables were manually coded: scoring side (left/right of field), attacking/defending designation, quarter (Q1–Q4), current score, play type (short corner/long corners), player position and opposition team. Score differential was calculated as the subject team’s score minus the opponent’s score at play initiation.

### 2.2. Data Preprocessing and Feature Extraction

Prior to analysis, extensive preprocessing and feature engineering were conducted to clean, organize and prepare the raw data for modeling. A key aspect of this process was the development of 13 team-level features listed below, designed to capture patterns of collective behavior in each play. Some of these features were directly observable, while others were engineered using game-specific heuristics.

1. Opposition (categorical): Opposing team;
2. Quarter (categorical): Game period Q1–Q4;
3. Time (continuous): Play duration in seconds (max seconds—min seconds);
4. Score_Differential (continuous): Goal differential at play start;
5. Player_Past23 (integer): Number of players involved past 23 m line.

A critical challenge was determining which players were “involved” in each play. The 23 m line in field hockey is a key tactical boundary that shapes where restarts, free hits, and long corners occur, making it one of the most important lines for both defending and attacking play. Thus, we used the 23 m line as a spatial threshold, where only players whose latitude coordinates placed them beyond the attacking 23 m line were included in feature calculations. This heuristic effectively filtered out defenders and non-participating forwards. The 23 m line latitude thresholds (−20.2911623 and −20.290753 for opposite end of pitch) were determined through inspection of the Bhubaneswar, India hockey center in Google Maps and the corresponding 23 m lines at the two opposite ends of the pitch. The pitch had a north–south orientation, allowing latitude to be used to operationalize these boundaries. Further to defining which players were ‘involved’, it is also important to note that in field hockey, there are no playing rules constraining how far or where a player may run; thus, the distance a player travels is not a prospective predictor of their play involvement.

The remaining features were derived from GPS data for players deemed involved in the play (Feature 5: Player_Past23):

6. Mean_Max_Velocity (continuous): Average of individual maximum velocities;
7. Mean_Max_Acceleration (continuous): Average of individual maximum accelerations;
8. Mean_Team_Velocity (continuous): Average of individual mean velocities;
9. Mean_DistanceTrav (continuous): Average distance traveled per player;
10. Corner_Positioning (continuous): Average distance of starting positions (first GPS point) from goal (fixed coordinates per side);
11. Closeness (continuous): Median inter-player distance during play, calculated using each player’s median position during the play;
12. Proportion_of_Goal Directional Linear_Runs (continuous): Proportion of linear versus nonlinear player trajectories.

To assess whether players ran in a consistent linear change in their position in the longitudinal direction of the pitch, we fit linear models (latitude ~ seconds) for each player’s trajectory. The adjusted R^2^ value quantified the strength of this linear relationship, with values > 0.8 classified as linear runs. The Proportion_of_Linear_Runs feature computed the team-level proportion of linear trajectories, hypothesized to indicate play structure versus chaos, under the assumption that coaching instruction emphasized linear movements towards the goal.

13. Mean_Player_Energy (continuous): Calculated as a continuous measure of the average movement energy characteristics of each play via wavelet analysis.

For each player, latitude and longitude time series were sampled at 10 Hz, transformed and analyzed separately using discrete wavelet transform (DWT) implemented in the wavelets R package. The DWT used the Daubechies least-asymmetric LA(8) filter, and a three-level decomposition was used [18,19]. The nominal Nyquist frequency was 5 Hz, and the third detail component (D3) represented an approximate frequency band of 0.625–1.25 Hz, corresponding to characteristic time scales of approximately 0.8–1.6 s. D3 was selected to capture relatively slower and more sustained fluctuations in the movement signal while limiting the influence of higher-frequency fluctuations. For each coordinate, the D3 component was reconstructed in the time domain, and energy was calculated as the mean squared amplitude of the reconstructed time series:

(1) Energy = ∑i=1NXi^2/N

where *N* is the number of observations and $X_{i}$ are reconstructed D3 time series values. The latitude- and longitude-based energy values were then averaged to yield player energy. Player-level values were subsequently averaged across all involved players in the play to derive Mean_Player_Energy. Higher values indicate greater movement-signal activity at the selected D3 time scale, reflecting more pronounced fluctuations in the recorded trajectory; lower values indicate smaller and smoother fluctuations. Because the measure was derived from latitude and longitude trajectories, it should be interpreted as a wavelet-based movement-variability measure rather than a direct physiological measure of metabolic or mechanical energy.

This process of feature construction results in a 63 by 14 data matrix, where each row corresponds to a play, and the columns comprise 13 features listed above and one binary outcome variable (successful play or not).

### 2.3. Modeling

The outcome variable, indicating whether the play was successful (coded as 1) or not (coded as 0), was modeled as a function of the extracted features using the random forest (RF) methodology implemented in R [14]. RF generates multiple decision trees on bootstrapped samples, aggregating predictions to reduce overfitting and improve accuracy. The RF model was fitted using 600 trees, with three predictors sampled at each split, and splits evaluated using the Gini impurity criterion.

Model performance was evaluated using repeated stratified 5-fold cross-validation with 10 repeats. Predictive performance was assessed using cross-validated prediction accuracy, area under the receiver operating characteristic curve (AUC), sensitivity, and specificity. Performance estimates were summarized across the 50 cross-validation resamples using their means and corresponding 95% confidence intervals. To provide an overall comparison with the no-information rate (NIR), one cross-validated prediction was obtained for each of the 63 unique plays by averaging the predicted Class1 probability across the 10 repeated cross-validation runs. Subsequently, a one-sided binomial test was used to test that the observed prediction accuracy was significantly greater than the NIR. This approach was used as the cross-validation resamples are not independent.

Feature importance was assessed within each cross-validation resample using the Random Forest Mean Decrease in Accuracy (MDA) measure. Importance values were calculated separately for each resampled training model and then averaged across the 50 resamples, with 95% confidence intervals calculated to describe variability in importance across resamples. For categorical predictors, including Opposition and Quarter, importance values for the individual factor levels were combined to provide an overall importance estimate for the predictor. Partial dependence plots were generated from the final model fitted to the full dataset to aid interpretation of the modeled relationships.

### 2.4. Software and Reproducibility

All analyses were conducted in R version 3.6.1. Packages/functions used were: randomForest, wavelets, geosphere, dplyr and ggplot2.

Custom functions were developed for feature extraction (Feature_Extractor22), visualization (Variable_Plotter), and classification wrapper functions, ensuring consistent train–test splits. In addition, a LonLot_Kilometers function was created that computed great-circle distances between GPS coordinate pairs using the Haversine formula, assuming Earth’s radius of 6378.145 km and ignoring elevation.

## 3. Results

Presented are the results of the Random Forest (RF) model, model performance, variable importance plots and partial dependence plots, finishing with the practical implications of the key findings.

### 3.1. Exploratory Data Analysis and Feature Distributions

Visual comparison of successful versus unsuccessful plays suggested several features showed promising separation, as explained below.

- Closeness: Successful plays exhibited ~8–15 m median inter-player distance versus more dispersed positioning in unsuccessful plays.
- Time: Successful plays clustered in the 3–6 s range, while unsuccessful plays showed a broader temporal distribution both less than and greater than this range.
- Player_Past23: Successful plays involved 9+ players more frequently.
- Mean_Max_Velocity and Mean_Max_Acceleration: Higher values associated with success.

### 3.2. Random Forest Classification Performance

The following results were obtained by fitting a Random Forest model using all 13 predictors with 5-fold cross-validation with 10 repeats:

- Mean Prediction Accuracy: 77.98% (95% CI: 74.93–81.02%);
- Mean Sensitivity: 57.2% (95% CI: 49.76–64.64%);
- Mean Specificity: 88.53% (95% CI: 85.78–91.28%;
- Mean AUC: 0.879 (95% CI: 0.849–0.908%);
- The no-information rate was 66.7% (*p*-value = 0.038 single aggregated cross-validated prediction).

**Figure 1:**
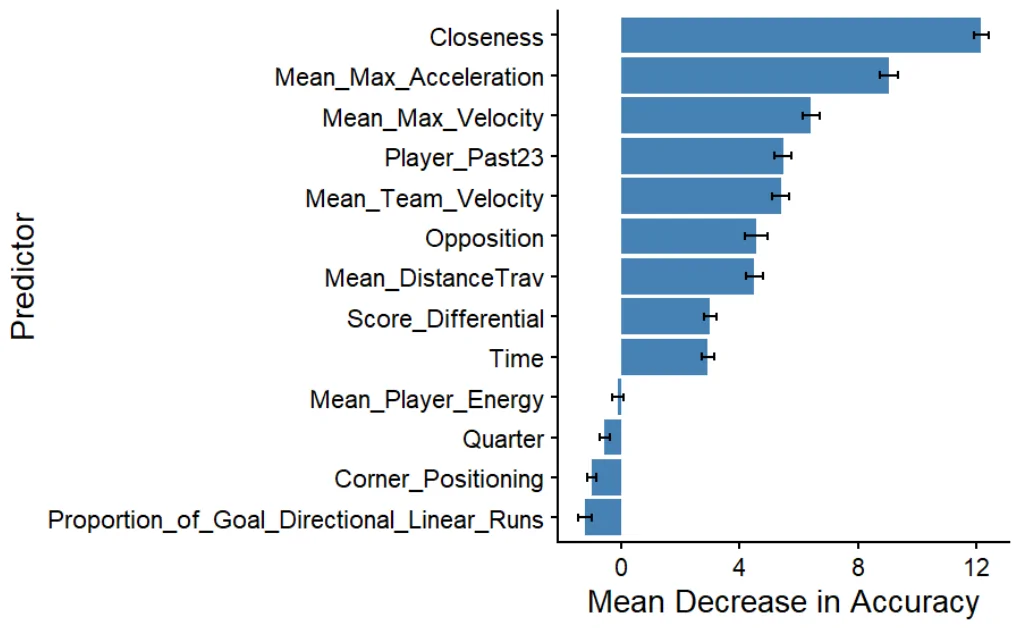
Variable importance plot with each bar representing Mean Decrease Accuracy and associated error bars for each predictor.

The feature importance analysis, presented in Figure 1, identified Closeness as the most important predictor, followed by Mean_Max_Acceleration, Player_Past23, Opposition, Mean_Max_Velocity, and Mean_Team_Velocity. Mean_Distance_Traveled, Score_Differential, and Time showed intermediate importance, while Mean Player Energy, Quarter, Corner_Positioning and Proportion_of_Goal_Directional_Linear_Runs demonstrated comparatively lower importance.

As a sensitivity analysis, removing Opposition from the original repeated five-fold cross-validation resulted in comparable performance (accuracy 77.1%, AUC 0.862), indicating that model performance was not substantially influenced by the inclusion of Opposition. Additionally, leave-one-match-out cross-validation was performed to account for match-level clustering. Performance metrics varied across the five held-out matches (n:10–16, NIR:0.5–0.88, Accuracy: 60–82%, Balanced Accuracy: 37–85%, AUC: 0.69–0.96, Sensitivity: 0.57–1, Specificity: 0–1), using the default 0.5 classification threshold per-fold tuning. Despite this variability, the relative importance of the principal predictors remained broadly consistent across analyses, suggesting that the identified relationships were robust within the available dataset.

### 3.3. Key Predictive Features Assessment

**Figure 2:**
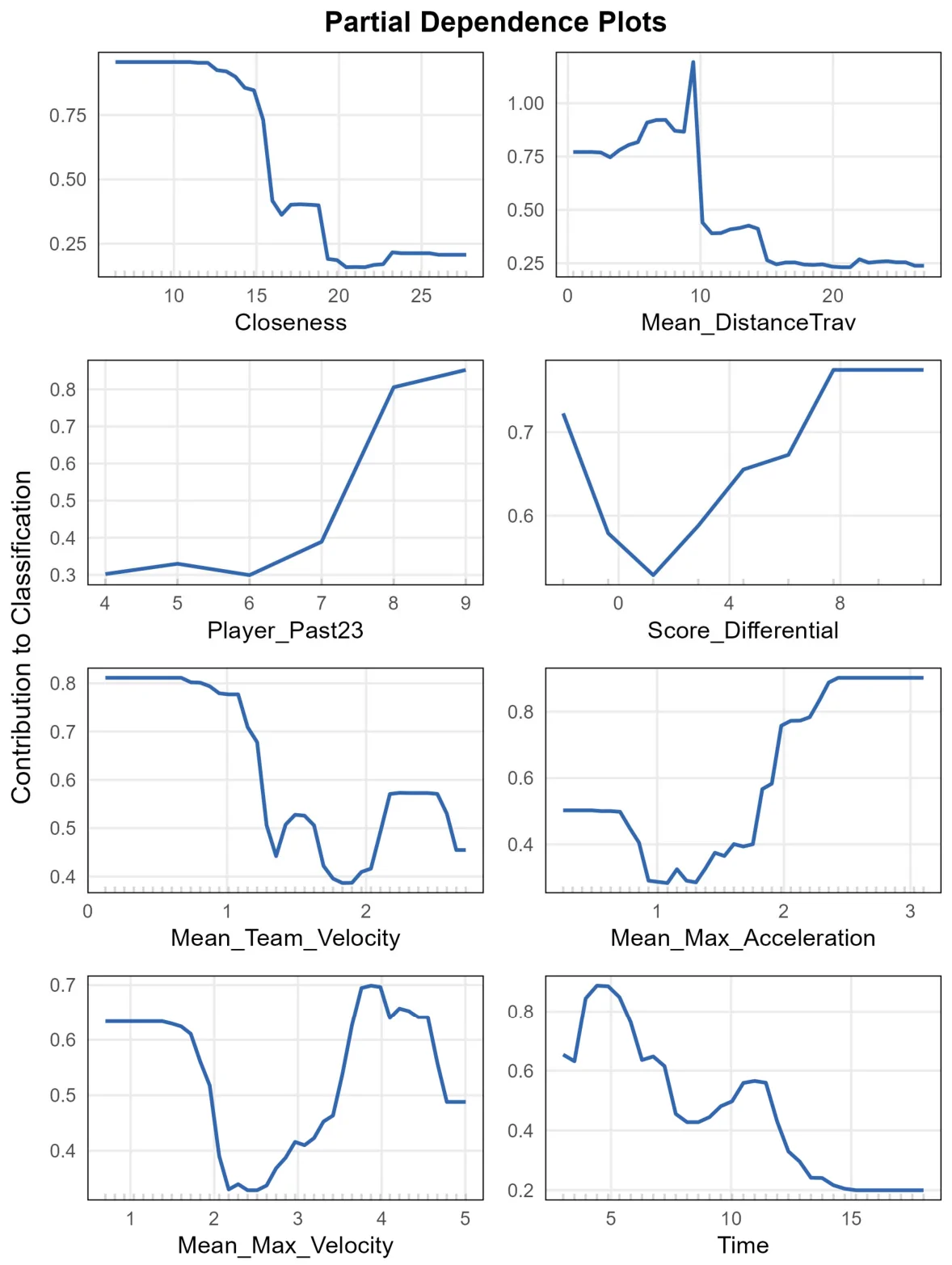
Partial dependence plots for the model fitted to the full data set. Each panel shows predicted probability of success (*y*-axis) across feature value range (*x*-axis), holding other features at mean values.

The partial dependence plots (Figure 2) illustrate how feature value ranges are associated with the likelihood of successful outcomes, when all other features are at their mean level. Interpreting each panel row in Figure 2, the following observations can be made regarding the feature values associated with success.

- Closeness: ~8–15 m;
- Mean_DistanceTrav: ~10 m;
- Player_Past23: ≥9 players;
- Time: 3–6 s;
- Mean_Max_Acceleration: >2.5 m·s^−2^;
- Mean_Max_Velocity: ~4.0 m·s^−1^;
- Score_Differential: U-shaped (high success when down by 2 or up by 6+);
- Mean_Team_Velocity: <1.0 m·s^−1^.

**Figure 3:**
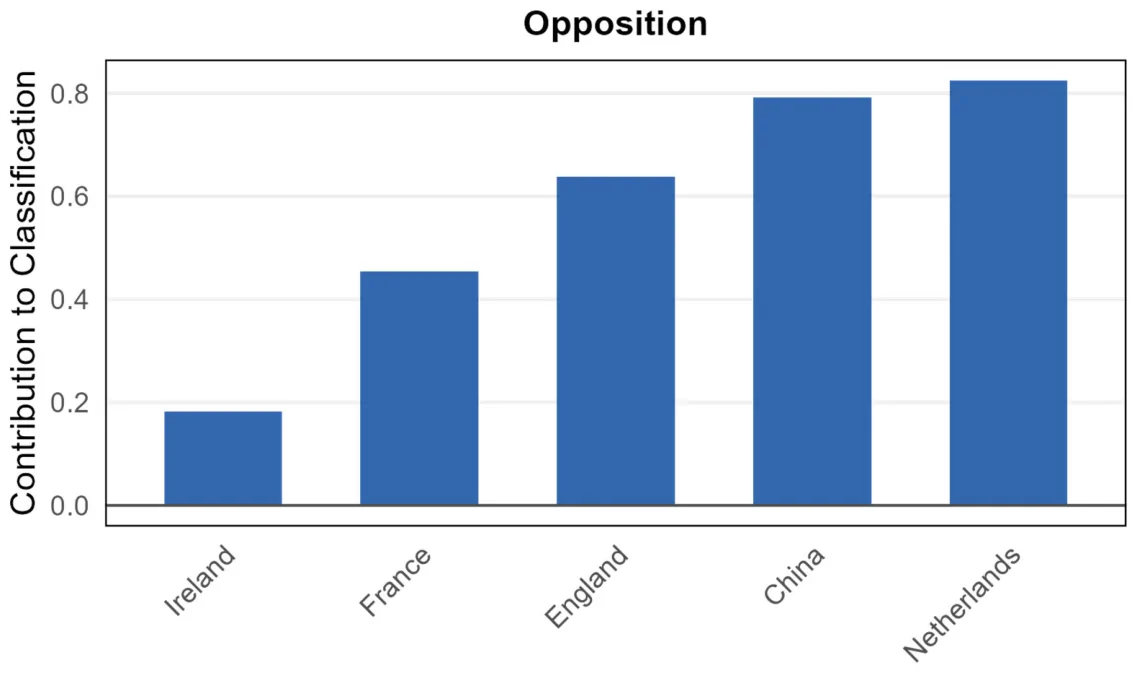
Partial dependency plot for categorical variable “Opposition”.

Consistent with the partial dependence plots in Figure 2, the analysis of the marginal contribution of the discrete feature ‘Opposition‘ to the classification outcome (Figure 3) indicates that matches against the Netherlands and China were associated with a higher likelihood of successful plays, whereas Ireland appears to present comparatively unfavorable conditions for generating successful plays.

## 4. Discussion

This study demonstrates that machine learning classifiers, particularly Random Forest, can effectively discriminate and characterize successful from unsuccessful attacking plays in field hockey using GPS-derived team features. The mean classification accuracy of 78% and AUC of 0.88 achieved across 5-fold cross-validation with 10 repeats with just 63 training examples and 13 features indicate that appropriate feature engineering can extract meaningful tactical patterns from spatial–temporal data despite small sample sizes typical of elite sports analysis. Furthermore, the data was limited to five countries, and the leave-one-match-out analysis represents a conservative evaluation that is sensitive to the small number and characteristics of the individual matches. However, additional data from other matches and countries are needed to fully assess model generalizability. Nevertheless, the principal predictors remained consistently important across all models and validation analyses.

The dominant importance of Closeness (~8–15 m inter-player spacing) suggests the study’s most actionable hypothesis that successful penalty corners may involve coordinated attacking structures with relatively tight positioning. This finding aligns with anecdotal field hockey tactical principles that closer positioning could support rapid passing, preserve attacking options, and create defensive overloads in dangerous areas, but these mechanisms were not measured and should not be treated as causal. Conversely, larger dispersed player positioning, particularly >15 m, may reflect a more fragmented attack, which allows more individual defending, but this also requires confirmation with tactical and opponent-position data. However, these findings are exploratory as player spacing was derived from GPS data, and device-specific positional error was not quantified. A reported 2.00 m SEM for total distance (reported using Catapult Vector S7 [20]) does not equal a 2.00 m SEM for position or spacing. Spacing uncertainty may combine error from both players’ devices and vary with satellite geometry/HDOP, satellite availability, device placement, speed and changes of direction. Therefore, observations near the 8, 12 and 15 m thresholds may be misclassified, and categories should remain provisional until inter-device positional accuracy is directly quantified for the device and protocol. Sensitivity analyses using plausible error bounds and validation under comparable field-hockey conditions are recommended before applying these thresholds to tactical decisions.

The temporal constraint (3–6 s duration) combined with moderate movement distances (~10 m per player) characterizes successful plays as explosive, decisive actions rather than patient play build-ups [21,22]. This specificity is consistent with established frameworks of reaction time and anticipation, wherein the number of discrete decisions required within a constrained time window is a critical determinant of successful performance, which can support coaches in designing training drills matching these temporal–spatial parameters. Research has consistently demonstrated that reaction time increases logarithmically with the number of stimulus–response alternatives, a relationship formalized as the Hick–Hyman Law [23,24]. In team sports, shorter plays reduce the number of decision cycles for attackers and defenders, emphasizing rapid perceptual-motor responses over deliberate tactical processing [25,26]. Abernethy [27] reported that skilled athletes offset this time pressure through anticipatory visual search, using advance postural cues to shorten effective decision time. It should be emphasized that any link between the 3–6 s window observed in the present data and the decision-cycle demands described by the Hick–Hyman Law is speculative; the present design does not permit a mechanistic account of this association, and this interpretation should be treated as a hypothesis for future testing rather than an established explanation. The relationship between decision-cycle demands and the temporal structure of successful team sport actions warrants further investigation and may in the meantime inform training drills that constrain task duration to reproduce match-like decision demands.

### 4.1. Comparison to Previous Sports Analytics Research

Our work extends previous computational sports science in several ways [2]. While video-based notational analysis and T-pattern detection have identified general patterns of play [8], these methods lack explicit success classification and quantitative spatial metrics. Heat map visualizations effectively display individual positioning but bias toward single-player analysis [5]. Self-organizing maps in basketball have classified play types [9], but success/failure classification outcomes from GPS positional data have only been reported in soccer [28].

Our contribution is the development and validation of team-level features, particularly inter-player distance metrics and wavelet-based movement energy, that capture collective behavior relevant to play outcomes. The Closeness metric offers a generalizable concept applicable across team sports: inter-player spacing likely varies by sport (tighter in hockey/soccer, looser in basketball/American football) but remains fundamental to coordinated attacks.

The methodological challenge of small datasets (n = 63) is addressed through careful feature engineering and cross-validation. While deep learning approaches dominate large-scale sports analytics, a Random Forest approach was used to characterize the relationships between the measured features and successful plays, as tree-based ensemble methods are well suited to capturing complex, nonlinear patterns in small datasets [13].

### 4.2. Practical Insights from Key Predictive Features

Across all models, Closeness consistently ranked as the most important feature. These team-level metrics, such as average distance between players during a play, captured tactical cohesion better than any individual player statistic, for example, Mean_Max_Velocity. The ~8–15 m inter-player spacing suggests successful plays involve coordinated clustering without overcrowding, likely facilitating rapid passing options while maintaining defensive pressure.

Mean_Max_Acceleration (>2.5 m·s^−2^) and Mean_Max_Velocity (~4.0 m·s^−1^) indicate successful plays require explosive movements and sprinting capacity. The counterintuitive negative relationship with Mean_Team_Velocity (<1.0 m·s^−1^) likely reflects that average velocity includes stationary positioning phases; explosive bursts (captured by max metrics) matter more than sustained running and likely also indicates a propensity for increased unstable patterning compared to a meta stable pattern that is easier to predict and defend against. Opposition effects suggest team-specific tactics may reflect favorable matchups or less effective defensive schemes from these opponents during the tournament.

Mean_DistanceTrav also emerged as a critical feature, with values around 10 m per player associated with successful play. Combined with the 3–6 s event duration, Time indicates successful plays are brief, explosive movements rather than prolonged build-ups.

### 4.3. Tactical Implications for Coaching

The reported exploratory computational findings may help explain successful attacking play and inform the following coaching recommendations:

- Maintain ~8–15 m Inter-Player Spacing: This “sweet spot” likely enables passing options without overcrowding. Training should emphasize spatial awareness and dynamic positioning to maintain effective distances as play evolves and reduce the risk of ball turnover.
- Demand Explosive Movements: High maximum acceleration (>2.5 m·s^−2^) and velocity (>4.0 m·s^−1^) requirements [29], suggest conditioning programs should prioritize anaerobic repeated sprint capacity over aerobic endurance for corner execution [30].
- Maximize Player Involvement: Getting 9+ players past the 23 m line, creating numerical advantages and overwhelming defenders with options, presenting a higher perceptual cognitive load to defenders, reducing the probability of a successful defensive response. Set-piece designs should incorporate off-ball runs to flood the attacking zone.
- Execute Rapidly (3–6 s): Successful corners are typically decisive and rapid. Defenders have less time to reorganize; prolonged plays allow defensive structure to form and decrease the cognitive decision-making load. Practice scenarios should emphasize quick decision-making and first-touch passing within a feedforward play structure that emphasizes prediction of the next pass rather than reaction.
- Opposition-Specific Preparation: Higher success rates between specific opponents indicate matchup dependencies. Video analysis combined with GPS patterns could identify exploitable defensive tendencies.

### 4.4. Limitations

Several limitations should be considered when interpreting the findings and their broader applicability. Importantly, we acknowledge that detailed sensor-level information such as the HDOP, satellite connection counts and positional error were unavailable and are therefore acknowledged as a limitation, particularly for interpreting small inter-player distance differences. Then, firstly, while the relatively small sample size (63 plays) provided adequate classification accuracy, it did limit the statistical power for subgroup analyses, such as comparisons between play types or opposition-specific patterns. Larger, multi-tournament datasets would improve robustness and enable the use of more data-intensive approaches, including deep learning. Second, moderate class imbalance may have influenced sensitivity–specificity trade-offs and inflated accuracy estimates. Future studies should consider balanced sampling strategies or stratified validation. Third, the binary definition of success combined outcomes with differing scoring probabilities, potentially obscuring tactical distinctions that could be addressed through multi-class classification. Generalizability is further constrained by using data from a single team and tournament, as team-specific tactics, opposition quality, and contextual factors may not extrapolate to other settings. In addition, elements of feature engineering, such as the 23 m involvement heuristic and wavelet parameter selection, involved subjective choices that warrant systematic evaluation. Fourth, the exclusive focus on attacking plays and 10 Hz GPS data may overlook defensive contributions and sub-second movement dynamics. Addressing these limitations through multi-team, multi-season data and refined sensing approaches will strengthen the proposed framework’s general application. Finally, the outcome plays were coded once by a single coder, and no duplicate or independent coding was undertaken. Consequently, intra-rater and inter-rater reliability could not be estimated. The reproducibility of the outcome classification therefore remains unverified, and potential coder-dependent classification error should be considered when interpreting the findings. This limitation does not establish that the classifications were inaccurate, but it prevents the reliability of the coding process from being empirically confirmed.

### 4.5. Future Directions

In practical terms, the established analytical workflow is readily transferable from offensive to defensive corner analysis. In this context, success criteria invert to emphasize preventing circle entries, blocking shots, or forcing turnovers, while the underlying feature-engineering and classification framework remains unchanged. Comparative evaluation of offensive and defensive patterns within this unified approach may therefore elucidate tactical asymmetries that are not evident when considering attacking plays alone. Extending the current binary success definition to a multi-class framework that distinguishes goals, shots on goal, and circle entries would enable more granular performance assessment. Furthermore, examining the temporal structure of play (e.g., inter-play intervals, number of plays per quarter or match) could facilitate investigation of the effects of physiological and cognitive fatigue, as well as psychological factors such as competitive pressure. Random Forest methods naturally support such multi-class classification, and analysis of outcome-specific partial dependence relationships may further refine tactical insights. Importantly, because the framework is grounded in general spatiotemporal principles rather than sport-specific heuristics, it is, in principle, applicable to other invasion sports in which coordinated team movement underpins performance outcomes. However, this transferability is likely to be partial rather than absolute. For example, the 23 m line represents a sport-specific constraint that may not generalize directly to other invasion games, particularly those with different scoring structures or defensive configurations, such as rugby (with distinct target zones) or Australian Football League (AFL), where there is no dedicated goalkeeper. When the nature, location, or presence of a scoring target changes, the associated task, environmental, and informational constraints are also altered, which may limit the direct transfer of spatial–temporal coordination principles derived from one sport context to another.

The wavelet-based Mean\_Player\_Energy metric, although excluded from the final Random Forest model due to negative predictive contribution, warrants further investigation given its potential to capture movement complexity beyond conventional kinematic measures. The discrete wavelet transform effectively distinguished erratic, high-frequency movement patterns from smoother, more linear trajectories; however, the current implementation likely underutilizes directional information by averaging latitude and longitude components. Future work should refine this metric through directional weighting of goal-oriented movements, validation against established load metrics (e.g., velocity zones and PlayerLoad™), evaluation of alternative mother wavelets, and extraction of additional frequency-domain features such as variance or entropy. Direction weighting in field hockey must account for the sport’s inherent biomechanical and spatial asymmetry. As an asymmetric, unilateral sport, stick grip, semi-crouched posture, and right-dominant side bending create structurally uneven musculoskeletal loading [31]. This is reflected in tactics, with most attacking circle entries occurring from the strong side [32]. Therefore, any directional weighting scheme must explicitly distinguish strong-side from weak-side approach trajectories, rather than treating all forward movement as equivalent. With appropriate optimization, wavelet-derived energy may provide a single, holistic indicator integrating velocity, acceleration, and directional change. Although entropy has been applied to accelerometer-derived signals in rugby league to quantify movement variability during tackling and resistance training contexts [33,34], its application as a frequency-domain feature of wavelet-decomposed positional tracking data remains unexplored and warrants investigation. Importantly, because this approach is grounded in general signal-processing principles rather than sport-specific heuristics, it is inherently transferable to other invasion sports using positional tracking data, provided that sport-specific movement constraints, including directional asymmetries, are accommodated within the weighting and feature-extraction framework. This transferability may support real-time load monitoring and decision-making applications across a range of team sport contexts [35].

The proposed framework, which combines spatial feature engineering from GPS-derived positional data with small-sample machine learning and tactically informed interpretation, is readily generalizable to other invasion sports such as association football, rugby, and lacrosse. While parameter values associated with success, such as inter-player spacing, event duration, and player involvement, are likely to be sport-specific, the underlying analytical pipeline remains transferable across contexts. Methods developed for retrospective analysis could also be adapted for real-time deployment by embedding feature-extraction routines within GPS hardware and associated software, enabling coaching staff to receive immediate feedback when predefined tactical thresholds are exceeded (e.g., excessive spacing, prolonged sequences, or insufficient player involvement). Integrating GPS-based metrics with video analysis would further strengthen interpretation and validation by linking quantitative spatial–temporal patterns to observable tactical behaviors, such as coordinated passing structures or rapid ball circulation. Finally, longitudinal application of this framework across multiple seasons would allow iterative refinement of tactical strategies and provide stronger causal evidence for whether adherence to model-derived recommendations lead to sustained improvements in performance outcomes.

### 4.6. Broader Implications for Computational Sports Science

This work demonstrates that dormant GPS data, ubiquitous in professional sports, holds untapped potential when paired with appropriate computational methods. The barrier is not hardware, such that GPS units are standard equipment, but rather problem framing and analytical capacity. Sports science would benefit from increased collaboration with data scientists and machine learning researchers to define answerable questions, engineer relevant features, and interpret results within domain contexts.

The wavelet-based energy metric exemplifies opportunities beyond traditional sports metrics. While velocity and acceleration are staples of load monitoring, wavelets offer holistic characterization of movement complexity. Similar innovations, such as applying signal processing, time series analysis, graph theory and topological data analysis, could revolutionize team sports analytics by quantifying previously qualitative concepts like “fluidity,” “cohesion,” or “chaos.”

Machine learning’s role in sports extends beyond prediction to insight generation. Variable importance plots and partial dependence curves translate black-box algorithms into interpretable tactical principles. This bidirectional flow, of domain expertise guiding feature engineering, while computational methods reveal unexpected patterns, represents ideal human–AI collaboration.

## 5. Conclusions

We developed and validated a machine learning framework for the characterization of successful field hockey plays from GPS tracking data. Random Forest achieved 78% five-fold 10-repeat cross-validated accuracy by leveraging novel team-oriented features, particularly player closeness (~8–15 m Inter-Player spacing) and player accelerations. Leave-one-match-out validation yielded variable predictive performance across held-out matches (accuracy 60–82%), reflecting the challenges of generalizing predictions to unseen matches. These findings provide actionable tactical intelligence for coaches while demonstrating that small sports datasets can yield robust insights through careful feature engineering. The methodology has the potential to generalize to other team sports, offering a template for extracting tactical knowledge from spatial–temporal tracking data. Future work should expand to defensive plays, refine wavelet-based load metrics, implement real-time tactical feedback systems and use larger datasets from additional competitions.

## Data Availability

The raw data supporting the analysis and conclusions of this article will be made available by the authors on request. Full source code is available on request.
